# Relationships of circular RNA with diabetes and depression

**DOI:** 10.1038/s41598-017-07931-0

**Published:** 2017-08-04

**Authors:** Guangjian Jiang, Yue Ma, Tian An, Yanyun Pan, Fangfang Mo, Dandan Zhao, Yufei Liu, Jia-Nan Miao, Yu-Jie Gu, Yangang Wang, Si-Hua Gao

**Affiliations:** 1 Diabetes Research Center, Beijing University of Chinese Medicine, Beijing, 100029 China; 2 Beijing University of Chinese Medicine Third Affiliated Hosiptal, Beijing, 100029 China; 3 Hebei Provincial Hospital of Traditional Chinese Medicine, Shi Jia Zhuang, 050011 China

## Abstract

Type 2 diabetes mellitus (T2DM) is closely related to depression; however, the exact molecular mechnisms of this association are unknown. Here, we investigated whether circular RNAs (circRNAs) in the blood are related to the occurrence of depression in patients with T2DM. Fourteen patients with T2DM and depressive symptoms, as assessed by the Self-Rating Depression Scale, were included in this study. Cutoff points of 44 (total coarse points) and 55 (standard score) were used to define depression. The Patient Health Questionnaire 9 was used for common mental disorders, and a score of 5 or more the cutoff for depression. Microarray assays and quantitative real-time reverse transcription polymerase chain reaction showed that 183 hsa-circRNAs were significantly upregulated, whereas 64 were downregulated in the T2DM with depression group (*p* < 0.05) compared with that in the T2DM group. Differentially expressed hsa-circRNAs could interact with microRNAs to target mRNA expression. KEGG pathway analysis predicted that upregulation of hsa-circRNA_003251, hsa-circRNA_015115, hsa-circRNA_100918, and hsa_circRNA_001520 may participate in the thyroid hormone, Wnt, ErbB, and mitogen-activated protein kinase signalling pathways. We speculate that differentially expressed hsa-circRNAs could help us to clarify the pathogenesis of depression in patients with T2DM and could represent novel molecular targets for clinical diagnosis and therapy.

## Introduction

Depression is a common psychiatric disorder with high morbidity and mortality, representing the second most common disease burden worldwide. Depression is estimated to have a heritability of about 37%. A number of investigative groups have reported epigenetic biomarkers (DNA methylation, microRNAs [miRNAs], and long noncoding RNAs [lncRNAs]) associated with the occurrence of depression; for example, some specific lncRNAs may have the ability to regulate major depression^1^. Many studies have shown that type 2 diabetes mellitus (T2DM) is closely related to the onset and progression of depression^2–4^, and several other reports have described the relationships between depression and T2DM^5–7^. However, the molecular pathophysiology, particularly the epigenetic regulation, of this association is still unknown.

A growing number of scientists have begun to focus on circular RNAs (circRNAs), which are noncoding RNAs that function as post-transcriptional regulators in various diseases. Recently, circRNAs have been reported to function as miRNA ‘sponges’ that naturally sequester and competitively suppress miRNA activity^8–11^. However, the roles of circRNAs in T2DM and their overall contributions to the pathogenesis of depression are still unclear.

Accordingly, in this study, we aimed to elucidate the associations of circRNAs with depressive disorder in patients with T2DM. Our findings provide important insights into the roles of circRNAs in patients of T2DM with depression.

## Results

### Profiles of circRNA expression in T2DM patients with depression

**Figure 1:**
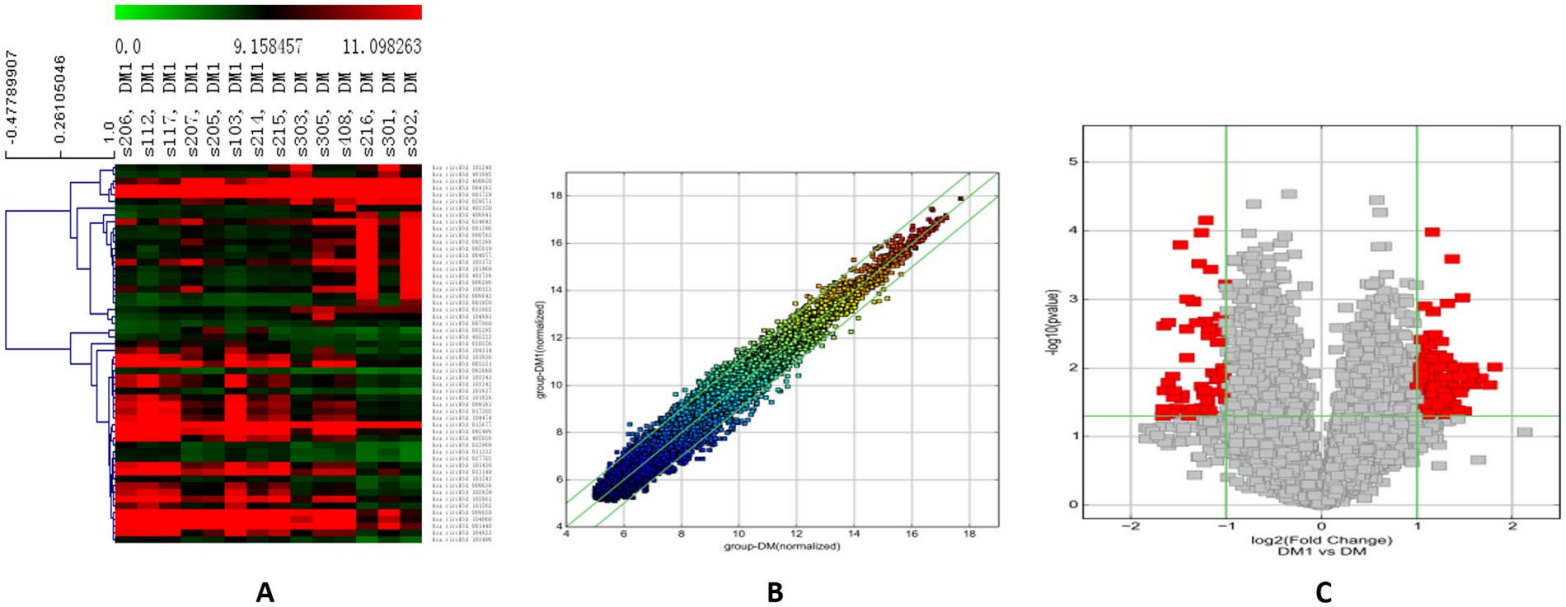
Differential expression of circRNAs in T2DM patients with depression. (**A**) Hierarchical clustering of circRNAs. Hierarchical clustering analysis of circRNAs that were differentially expressed between T2DM (DM) and T2DM with depression (DM1) groups; each group included seven individuals (fold change >2; *P* < 0.05). (**B**) Scatter plot of circRNAs. Scatter plots were created to assess variations in circRNA expression between DM and DM1 samples. The values corresponding to the X- and Y-axes are the normalised signal values. (**C**) Volcano plots of circRNAs. Volcano plots were constructed by fold-change and *p* values. The vertical lines correspond to *P* values, and the horizontal lines represent log2 (fold change) up- and downregulation between DM1 versus DM samples. The red points show the significantly differentially expressed circRNAs.

Overall, 247 circRNAs were differentially expressed between the T2DM (DM) and T2DM with depression (DM1) groups according to microarray analysis (Fig. 1). Of these, 183 circRNAs were significantly upregulated, and 64 were downregulated among samples from T2DM patients with depression.

### Validation of differentially expressed circRNAs by qRT-PCR

**Figure 2:**
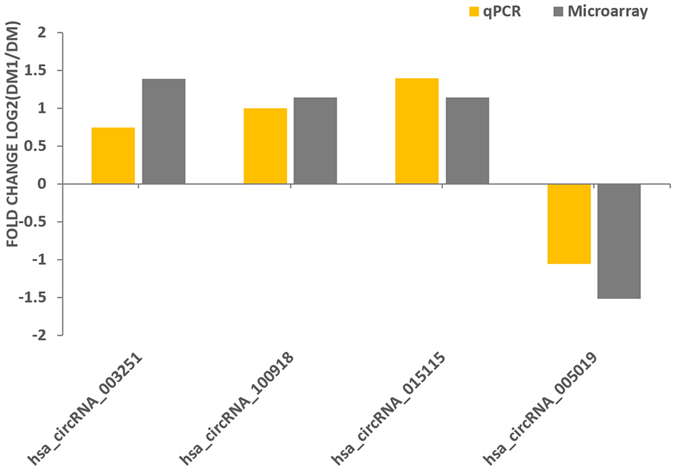
Validation of the four differential expression circRNAs. The expression levels of the following hsa _circRNAs were analysed by qRT-PCR: hsa_circRNA_003251, hsa _circRNA_015115, hsa _circRNA_100918, and hsa_circRNA_005019. The heights of the columns represent the fold changes (log2 transformed) computed from the qPCR and microarray data.

To validate the circRNA microarray results, we selected four circRNAs that exhibited significant changes in expression among the differentially expressed circRNAs, and then performed qPCR to analyse the changes. Importantly, the qRT-PCR results of hsa-circRNA_003251, hsa-circRNA_015115, hsa-circRNA_100918, and hsa-circRNA_005019 were consistent with the microarray data, verifying the reliability of the circRNA profile (Fig. 2).

### MiRNA-gene prediction and annotation

We identified circRNA/miRNA networks by using Target Scan and Miranda softwares. The top six predicted target miRNAs of hsa-circRNA_015115 were *hsa-miR-6760-3p*, *hsa-miR-761*, *hsa-miR-6728-3p*, *hsa-miR-5693*, *hsa-miR-298*, and *hsa-miR-367-5p*. The top four predicted target miRNAs of hsa-circRNA_005019 were *hsa-miR-298*, *hsa-miR-3155a*, *hsa-miR-1306-5p*, and *hsa-miR-4460*. The top five predicted target miRNAs of hsa-circRNA_003251 were *hsa-miR-4753-3p*, *hsa-miR-301a-5p*, *hsa-miR-3191-5p*, *hsa-miR-6805-5p*, and *hsa-miR-761*. Additionally, the top five predicted target miRNAs of hsa-circRNA_100918 were *hsa-miR-33b-5p*, *hsa-miR-33a-5p*, *hsa-miR-148a-5p*, *hsa-miR-502-5p*, and *hsa-miR-891b*. Interestingly, *hsa-miR-761* and *hsa-miR-298* were found to be targets of hsa-circRNA_003251, hsa-circRNA_005019, and hsa-circRNA_015115. All of the above targets and other important target genes were verified by biological experiments as follows.

### Prediction of circRNAs related to depression and target genes

**Figure 3:**
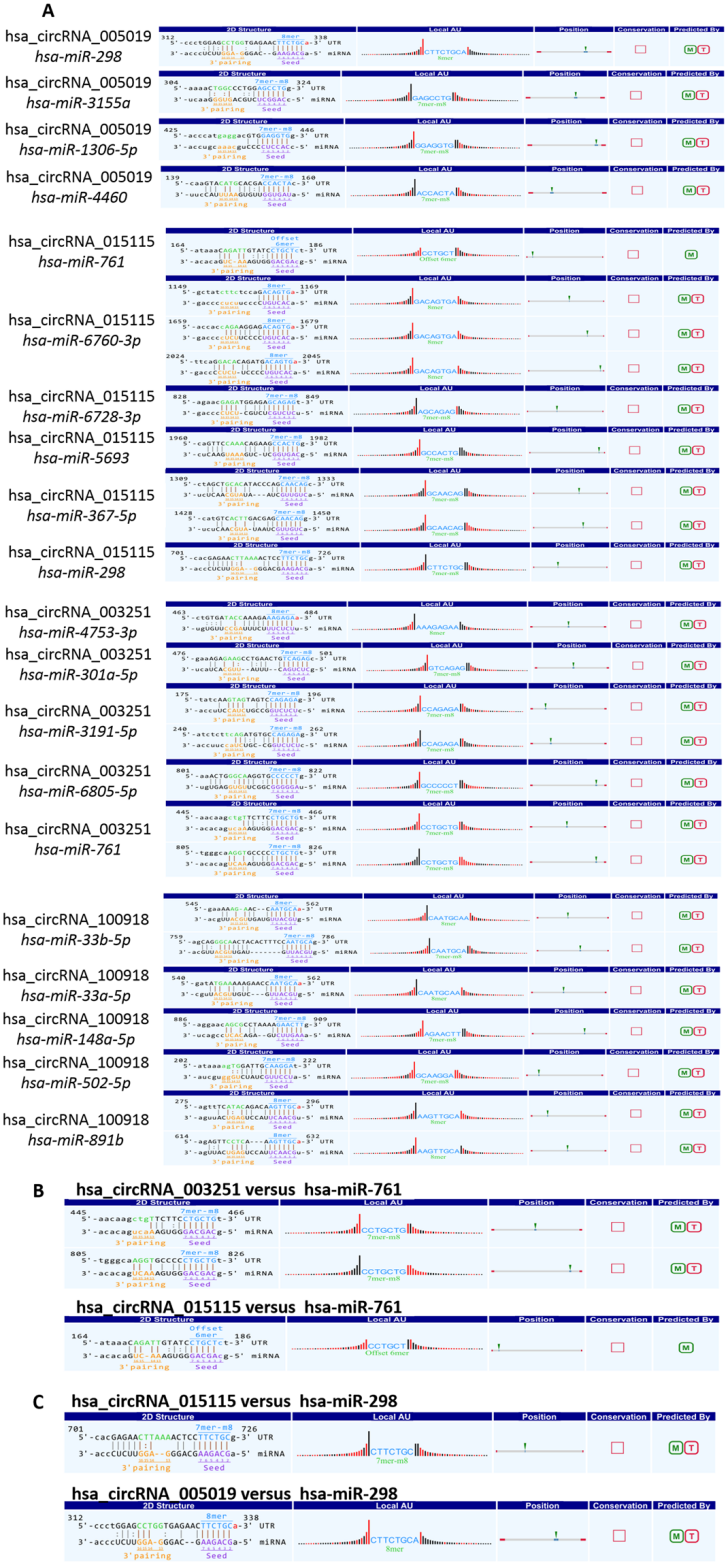
Relationships between four circRNAs and their target microRNAs. (**A**) MiRNAs targeted by hsa-*circRNA*_005019, hsa-*circRNA*_015115, hsa-*circRNA*_003251, and hsa-*circRNA*_100918. (**B**) *hsa-miR-761* matched with hsa-*circRNA*_003251 and hsa-*circRNA*_015115. (**C**) *hsa-miR-298* matched with hsa-*circRNA*_015115 and hsa-*circRNA*_005019.

The target miRNAs of hsa-circRNA_015115, hsa-circRNA_003251, hsa-circRNA_100918, and hsa_circ_0005019 were identified and ranked based on mirSVR scores. Specific details of the molecular interactions between the above circRNAs and their target miRNAs are depicted in Fig. 3.

**Figure 4:**
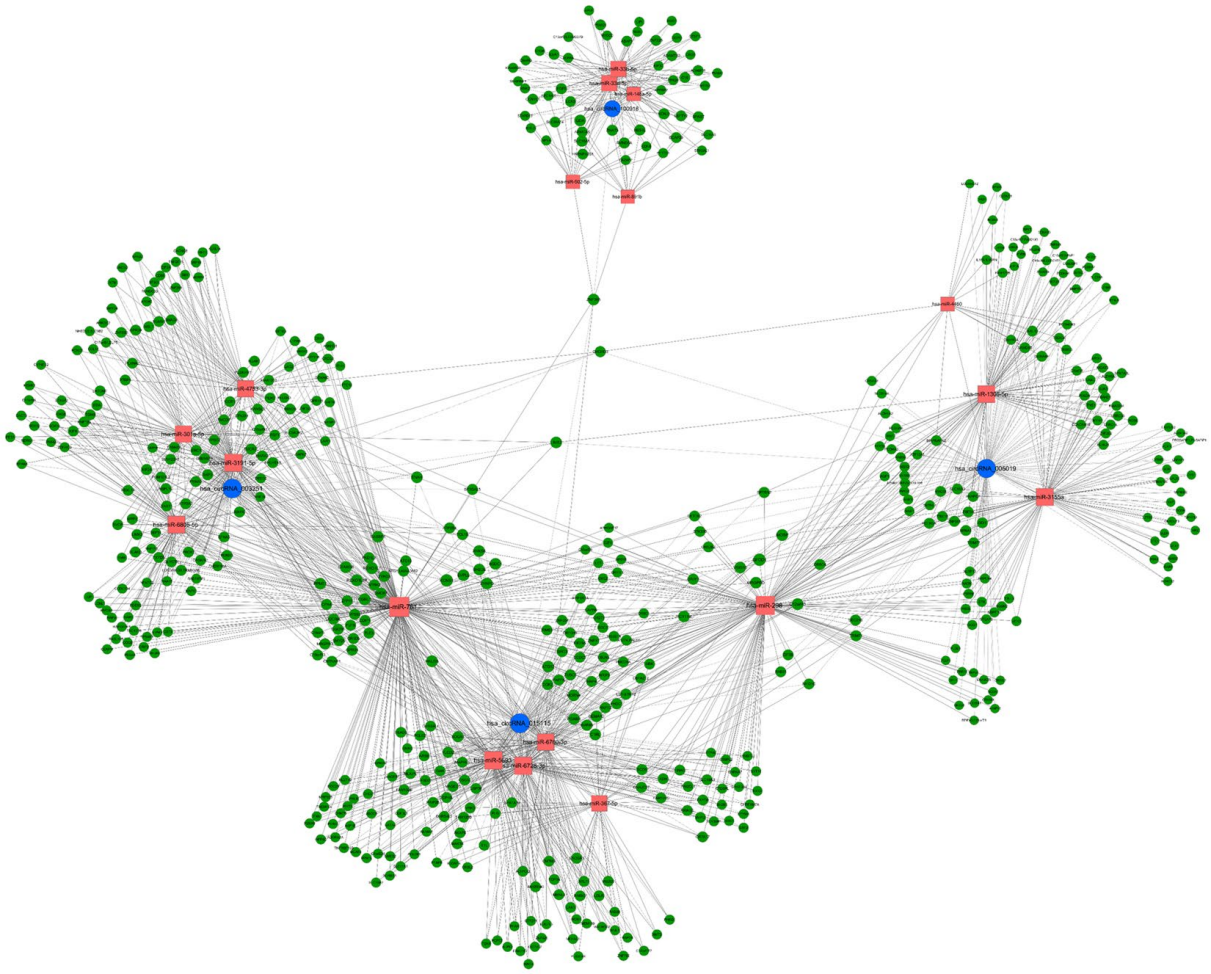
CircRNA/miRNA/mRNA network. Blue circle nodes: circRNAs; red square nodes: miRNAs; green circle nodes: mRNAs.

To predict the circRNA targets and target genes related to depression, we constructed circRNA/miRNA/mRNA co-expression networks according to the microarray results, common miRNA-binding circRNAs, and mRNAs. Eighteen miRNAs and 529 mRNAs were found to have interactions with four circRNAs, as predicted by TargetScan and Miranda (Fig. 4). These data indicated the tight correlations and regulatory relationships among these genes.

### Enrichment analysis of circRNA-targeted differentially expressed genes

**Figure 5:**
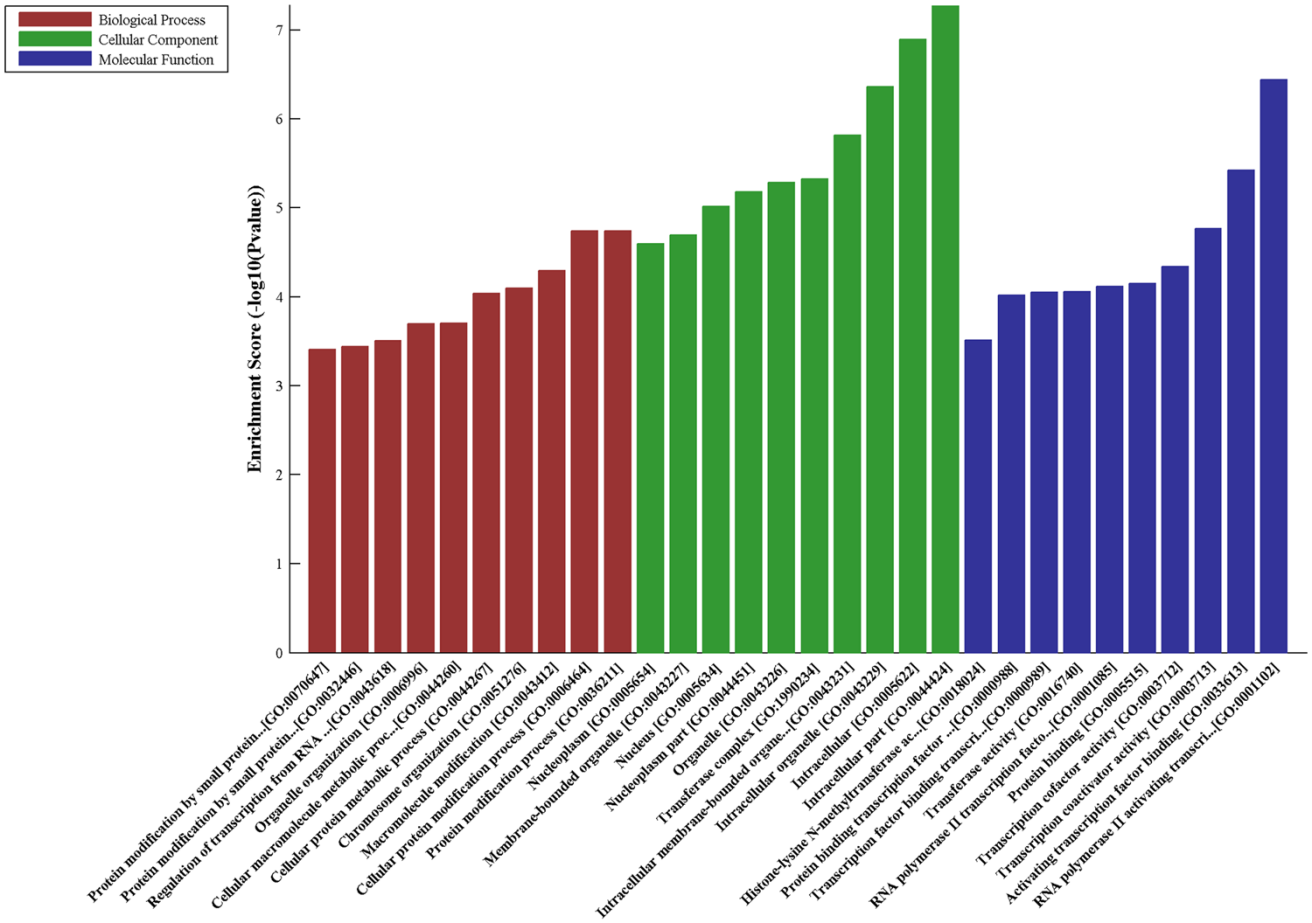
GO enrichment for circRNA-targeted mRNAs upregulated in samples from the DM1 group.

GO analysis was applied to further predict and clarify the functions of circRNAs. Upregulated target mRNAs in patients with T2DM with depression were analysed to determine differences in molecular function, biological processes, and cellular components (Fig. 5). The target mRNAs were found to play important roles in the pathogenesis of diabetes mellitus and depression, including histone methylation, cellular metabolic processes, and DNA damage responses, as well as macromolecular functions involved in chromatin regulation, including SUMO ligase activity, histone methyltransferases, mitogen-activated protein kinase (MAPK) activity, and transcription factor binding.

**Figure 6:**
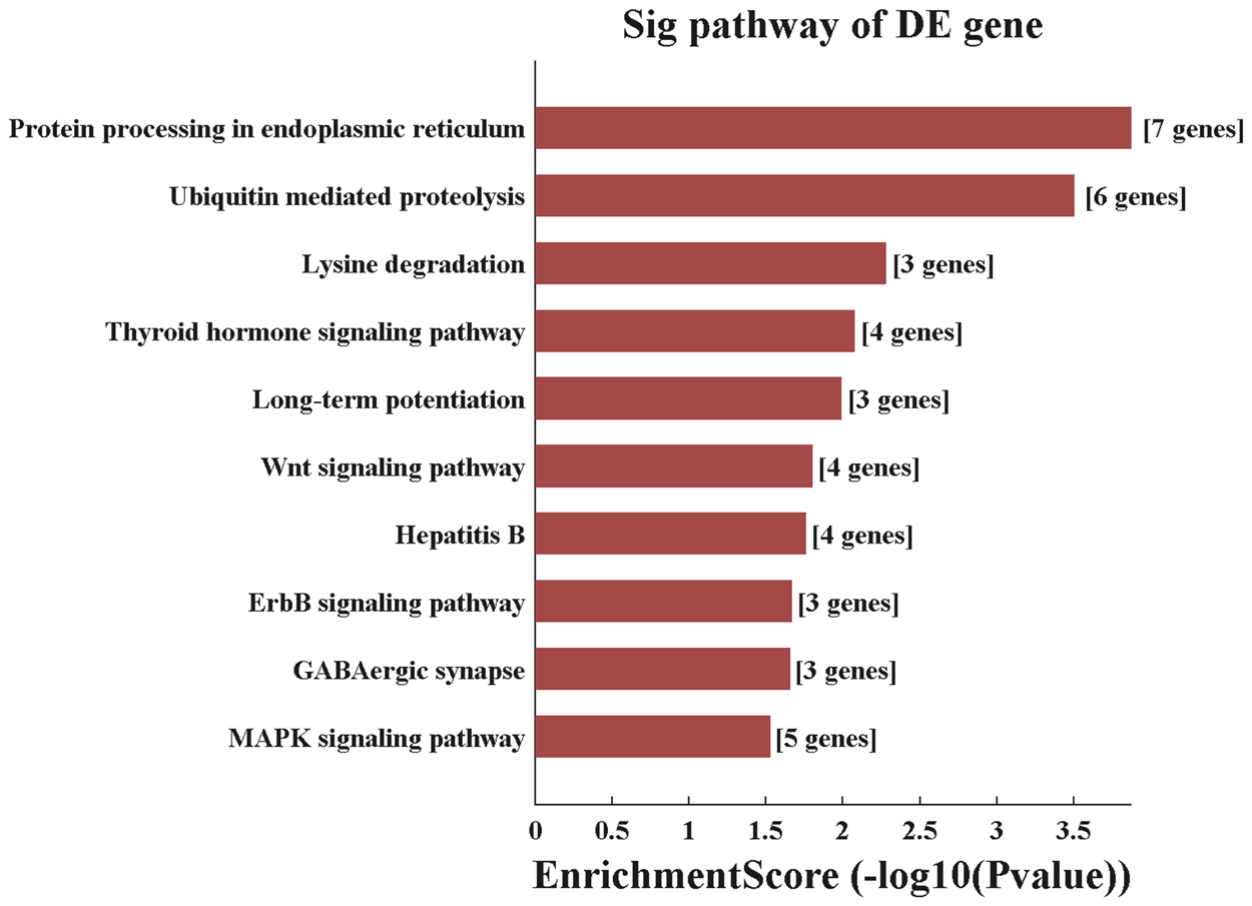
Annotated pathways significantly targeted by circRNAs.

Upregulated KEGG pathways of target mRNAs in the DM1 group were found to participate in ubiquitin-mediated proteolysis, GABAergic synapse function, lysine degradation, long-term potentiation, protein processing of the endoplasmic reticulum, and the thyroid hormone, Wnt, ErbB, and MAPK signalling pathways (Fig. 6).

## Discussion

Approximately 10% of patients with T2DM also exhibit depression, and about two out of every five patients with T2DM have alexithymia^12, 13^. Thus, there is an increased risk of depression in patients with T2DM. Moreover, patient outcomes are generally worsened by the co-occurrence of depressive disorders and T2DM. Accordingly, treating depression is thought to have beneficial effects on patients with T2DM. However, the exact molecular mechanisms through which depression is associated with T2DM are still unclear.

In the present study, we identified many circRNAs that were aberrantly expressed in T2DM patients with depression compared with those without depression. Depression is a complex disease induced by the correlation of epigenetic inheritance and environmental effects, including circRNAs, affecting certain specific brain circuits and nervous system components. In our study, the different circRNA expression profiles were screened by the circRNA array technique and verified again by qRT-PCR. The upregulated target gene *DCP2* (hsa_circRNA_001520) could degrade mRNAs as a decapping enzyme, which has been shown to play a major role in neurological development and mental retardation^14, 15^. Another upregulated target gene *PICALM* (hsa_circRNA_100918) was found to be associated with disorders of glucose metabolism. Moreover, this gene could modulate autophagy and was related to the pathology of Alzheimer’s disease as a genetic risk factor^16, 17^. Accordingly, we speculate that PICALM may affect the occurrence of depression in patients with T2DM via a number of molecular mechanisms, particularly the cellular autophagy pathway.

The downregulated target gene *CHSY1* (hsa_circRNA_005019) encodes the chondroitin N-acetyl galactosaminyl transferase family; the protein has glucuronyl and galactosaminyl transferase activity and participates in many metabolic processes. *CHSY1* aberrations have been observed in patients displaying mental retardation with or without dysmorphic features^18, 19^. Another target gene, *CSGALNACT1* (hsa_circRNA_001781), which was downregulated in patients with T2DM with depression, was reported to be related to the antidepressant response^20^. The above results suggest that T2DM may be associated with the onset of depression and has an epigenetic basis. Moreover, the functions of other target genes are related to many important biological processes, such as histone methylation, indicating that circRNAs could participate in epigenetic control by sequestering miRNAs like a sponge.

Pathway knowledge can provide disease marker information that is crucial to diagnosis, drug choice, and patient treatment. In our study, the target genes of circRNAs were characterised by pathway analysis. The long-term potentiation, GABA-B receptor, thyroid hormone, and Wnt signalling pathways, which are critical for determining the specific pathophysiological mechanisms of disease, were predicted to have strong relationships with the target genes. Our present findings suggest that dysregulation of thyroid hormone metabolism and changes in thyroid status may be involved in the occurrence of depression. Many studies have shown that mood can be influenced by the levels of thyroid hormones; for example, Henley reported that thyroid hormones could promote signalling pathways in the brain^21^. Consistent with these outcomes, our results also indicated that thyroid hormone signalling pathways may play a crucial role in the development of depression via the cerebrospinal axis.

The Wnt/β-catenin signal transduction pathway is closely related to neurogenesis in the adult hippocampus. Nerve regeneration is critical to the occurrence, development, and treatment of depression, suggesting that the Wnt signal pathway may be correlated with the progression of depression by activating different neuroprotective mechanisms. In contrast, the representative Wnt signalling pathway plays well-known functions in metabolic syndrome, particularly T2DM^22^. T2DM and depression could be bidirectionally regulated by the Wnt signal pathway, based on a variety of large-scale genome-wide association studies^23^, providing insights into the specific pathological mechnisms of T2DM with depression. Additionally, ubiquitin-mediated proteolysis and long-term potentiation were also dysregulated, similar to the findings of other studies^24, 25^. These above findings indicated that circRNA target genes were associated with known functional pathways relevant to T2DM with depression; therefore, the related circRNAs were assumed to have pivotal roles in the etiology of depression. Further studies are needed to determine the exact functions of these circRNAs. Moreover, the specific circRNAs identified from the two groups could act as biomarkers and potential therapeutic targets for future interventional studies, diagnosis, and treatment of T2DM with depression.

hsa-circRNA _005019, hsa-circRNA-015115, hsa-circRNA_003251, and hsa-circRNA_100918 may function as miRNA “sponges.” To counteract their target miRNAs, we constructed a circRNA/miRNA/mRNA network that provided information regarding the potential associations between circRNAs and their target genes. These networks provided an important reference value for studying the interactions of differentially expressed circRNAs and their potential targets. Collectively, our data indicated that 247 circRNAs were dysregulated (183 upregulated, 64 downregulated) in T2DM patients with depression. These observed changes have biological effects, indicating that circRNAs are key regulators of gene expression.

In this experiment, we found that hsa_circRNA_015115 and hsa-circRNA_003251 may function as “sponges” by competitively binding *hsa_miR-761* in the predicted circRNA/miRNA/gene network. Additionally, *hsa_miR-761* was confirmed to regulate the mitochondrial network and facilitate the induction of learning and memory^26, 27^. Overexpression of *hsa_miR-761* could inhibit the p38 MAPK signal pathway^28^. Consequently, we speculated that *hsa_miR-761* may participate in the pathogenesis of depression through regulation of target genes and then reflect a series of biological functions and pathways, such as the MAPK signal pathway. However, further studies are needed to determine the exact mechanisms.

In the present report, functional analysis and prediction of naturally expressed circRNA profiles were carried out in patients with T2DM with or without depression for the first time. Our results demonstrate that circRNAs will become new targets for physicians to detect, confirm, and treat depressive disorders in patients with T2DM. Deciphering the precise molecular mechanisms through which circRNAs affect depression and diabetes will require more samples and in-depth studies to improve our understanding of the pathophysiology of depression in patients with T2DM and facilitate the exploration of new potential therapeutic targets in the future.

## Materials and Methods

### Patient samples

Ethylenediaminetetraacetic acid (EDTA)-treated whole blood samples from seven patients with T2DM (four women and three men, age range: 48–65 years, average age: 58.14 years) with current major depressive episodes (the DM1 group) and seven patients with T2DM (four women and three men, age range: 41–69 years, average age: 58.28 years) without major depressive episodes (the DM group) were collected after obtaining informed consent. This study was carried out according to the guidelines of the Declaration of Helsinki, and the protocol was approved by the Ethics Committee of Beijing He ping li Hospital. All patients were evaluated by laboratory tests and semistructured interviews to assess the clinical features of T2DM. Patients’ depression statuses were evaluated using the Self-rating Depression Scale (SDS) and Patient Health Questionnaire 9 (PHQ9), as shown in Table 1. Cutoff points of 44 (total coarse points) and 55 (standard score) were used to define depression for the SDS, whereas a score of 5 or more was used as a cutoff to define depression for PHQ9.

**Table 1:** SDS and PHQ9 scores in the DM1 and DM groups.

Patient ID	Age (years)	Sex	Total coarse points	Standard score	PHQ-9
**DM1**					
117	55	Female	49	61	12
206	60	Female	49	61	17
112	65	Male	52	65	13
103	48	Female	46	57	6
207	64	Male	53	66	23
205	62	Male	44	55	5
214	53	Female	47	58	6
**DM**					
215	41	Male	25	31	2
305	62	Female	29	36	4
408	69	Male	31	38	0
303	51	Female	26	32	3
301	61	Female	36	45	3
216	62	Female	33	41	4
302	62	Male	37	46	1

### RNA isolation, circRNA labelling, and hybridisation

Total RNA was isolated from the 14 samples, using TRIzol reagent (Invitrogen, Carlsbad, CA, USA) according to the manufacturer’s instructions. Total RNAs were digested with RNase R (Epicentre, Inc., Madison, WI, USA), then linear RNAs were removed, and circRNAs were enriched. The fluorescent complementary RNA (cRNA) was amplified and transcribed by enriched circRNAs, using a random priming method (Arraystar Super RNA Labeling Kit; Arraystar, Rockville, MD, USA). In the experiment, 1 μg RNA was used for labelling. The labelled cRNAs were hybridised onto the Arraystar Human circRNA Array v2 (8 × 15 K; Arraystar). After having washed and fixed the slides, the hybridised arrays were scanned by an Agilent Scanner G2505C. Array images were acquired using Agilent Feature Extraction software (version 11.0.1.1; Agilent Technologies, USA). The R software limma package was used to perform quantile normalisation and subsequent data processing. The circRNAs for which at least seven out of 14 samples had flags in “P” or “M” (defined by GeneSpring software) were kept for subsequent differential analyses.

### Quantitative real-time PCR (qRT-PCR) validation

qRT-PCR was performed to verify the microarray result. Purified total RNAs isolated from 14 samples were reverse-transcribed into cDNA according to the manufacturer’s instructions. The reverse transcriptase reactions contained 1.5 µg RNA, 0.5 μg/μL random primers (N9, 1 μL), 2.5 mM dNTPs mix (1.6 µL), 5× first-strand buffer (4.0 μL), 0.1 M dithiothreitol (DTT, 1 µL), RNase inhibitor (0.3 µL), and Superscript III RT (0.2 µL). All data were normalised to data for β-actin to calculate relative circRNA concentrations. The genes and primers are shown in Table 2.

**Table 2:** Specific circRNA primers for quantitative PCR analysis.

Primer name	Sequence	A T (°C)	PS (bp)
β-Actin (H)	F: 5′-GTGGCCGAGGACTTTGATTG-3′	60	73
	R: 5′-CCTGTAACAACGCATCTCATATT-3′		
hsa_circRNA_003251	F: 5′-GCAGCAAATCTAGTCGAAGCA-3′	60	136
	R: 5′-ACAGAAACCTGAAATCGTCCC-3′		
hsa_circRNA_015115	F: 5′-CTGATCCCAGGTGCAAGGTAT-3′	60	111
	R: 5′-CAGTGTCATTCCAACAGATTGTATTA-3′		
hsa_circRNA_100918	F: 5′-GGACACCTACTCATGAAATGTTTG-3′	60	91
	R: 5′-TCAAATAAACTGTCTGCCAACTG-3′		
hsa_circRNA_005019	F: 5′-GCAGTGTGTCTGGTCTTATGAGAAC-3′	60	79
	R: 5′-TCAGAACCCTCACTTGAGAAGAACT-3′		

AT, annealing temperature; PS, product size.

### Target prediction and functional analysis

CircRNAs were selected according to the circRNA profiling data. The interactions of circRNAs and mRNAs with miRNAs were calculated using the biological algorithm of a custom miRNA target prediction program. Arraystar composed the custom software using R language, which was based on Target Scan and Miranda software. An mRNA/miRNA/circRNA network was constructed according to the common target miRNAs of the circRNAs and mRNAs. According to gene ontology enrichment analyses and pathway enrichment of Kyoto Encyclopedia of Genes and Genomes, we selected target mRNAs to construct the network according to our previous microarray data and to annotate the potential functions of the target genes^29^.

### Statistical analysis

Significant differences in the circRNA microarray results between two groups were estimated by t tests (fold change >2; *P* < 0.05). qRT-PCR results were tested by Student’s t tests, and *P* values of less than 0.05 were considered to indicate statistically significant differential expression.

### Data availability statement

The data in this article is available.
